# Causal relationships of mental diseases and thyroid diseases based on a Mendelian randomization study

**DOI:** 10.1097/MD.0000000000038223

**Published:** 2024-05-31

**Authors:** Xiang Fang, Cuiping Wu, Wenjing Ding, Dandan Xu, Zhangxia Shi

**Affiliations:** a Hefei Second People’s Hospital, Hefei, China; b The First Affiliated Hospital of China University of Science and Technology (Anhui Provincial Hospital), Hefei, China; c The Second Clinical Medical School, Anhui University of Chinese Medicine, Hefei, China.

**Keywords:** causality, genome-wide association research, Mendelian randomization, mental diseases, thyroid diseases

## Abstract

Evidence from observational researches have suggested that mental diseases are able to affect thyroid diseases. However, the causal relationship between mental diseases and the risk of thyroid diseases still remains unclear. Herein, we conducted a two-sample Mendelian randomization (MR) statistical analysis method to assess the causality between mental diseases and thyroid diseases. Initially, publicly available genome-wide association studies summary data were leveraged to obtain single-nucleotide polymorphisms based on set parameters. Subsequently, a two-sample MR was utilized to analyze causal relationships between mental diseases (Alzheimer disease, bipolar disorder, major depressive disorder, Parkinson disease, schizophrenia) and thyroid diseases (hyperthyroidism/thyrotoxicosis, hypothyroidism) with removing outliers based on MR-PRESSO method. Finally, 8 regression MR methods (inverse variance weighted [IVW], IVW fixed effects, c, MR Egger, weighted median, penalized weighted median, simple mode, weighted mode) were performed to evaluate bias and effectiveness, of which IVW was considered as the primary method. Our results demonstrated that most of mental diseases have no causal relationships with thyroid diseases except bipolar disorder and hyperthyroidism/thyrotoxicosis based on IVW method [odds ratio: 0.999, 95% confidence interval: 0.998–1.000, *P* = .028], and bipolar disorder and hypothyroidism based on IVW method [odds ratio: 0.997, 95% confidence interval: 0.995–0.999, *P* = .002]. Then we subsequently conducted a consistent robustness analysis to assess heterogeneity and horizontal pleiotropy. Our method reports causal relationships exist mental diseases and the risk of thyroid diseases. Subsequent researches are still warranted to determine how mental diseases influence the development of thyroid diseases.

## 1. Introduction

The thyroid disease (TD) is a common, constantly increasing disorder affecting up to 3/1000 patients in Europe per year.^[1]^ Thyroid dysfunction disorder, such as hyperthyroidism, hypothyroidism, and thyrotoxicosis, are well-known risk factors of reason, and they have an important influence on patient life.^[2]^ Hyperthyroidism is a pathological disease that indicates a condition as a result of an exaggerate production of thyroid hormone (TH).^[3]^ It is characterized by normal or high thyroid radioactive iodine uptake^[4]^ and Graves’ disease is the most common cause.^[5]^ For hypothyroidism,^[6]^ it potentially devastates health consequences that affect populations all over the world,^[7]^ which refers to the common pathological condition of TH deficiency. Overt or clinical primary hypothyroidism is considered as thyroid-stimulating hormone (TSH) concentrations above the normal range and free thyroxine concentrations below the normal range. Mild or subclinical hypothyroidism^[8]^ is defined by TSH concentrations above the reference range and free thyroxine concentrations in the common range. Thyrotoxicosis is a disease with additional TH, which can be related to hyperthyroidism or can also occur in the absence of increased TH secretion.^[9]^ Similar to hyperthyroidism, Graves’ disease is also the common causal of thyrotoxicosis.^[10]^

Mental diseases (MDs) is becoming increasingly common in modern populations all over the world,^[11]^ and it contains Alzheimer disease, bipolar disorder, major depressive disorder (MDD), Parkinson disease, and schizophrenia. Several literature have reported causal associations between MDs and TDs. In,^[12]^ a systematic review was conducted to determine the association between thyroid function and Alzheimer disease. Evidence has been discovered that changes in THs levels, divided into thyroxine (T4) and triiodothyronine, and hypothyroidism/hyperthyroidism are related to learning and memory impairment and development of Alzheimer disease.^[13]^ Results from^[14]^ demonstrated that there is a higher popularity of thyroid diseases in people with mental disorders (like: bipolar disorder and schizophrenia) and thyroid functions were significantly different between depression and bipolar disorder of patients with clinical significance.^[15]^ A recent study has reported that free THs concentrations are related to MDD and lead to final clinical results, and it can be more effective to treat MDDs with triiodothyronine.^[16]^ The systematic review and meta-analysis method in^[17]^ confirmed that there was an important causal link of both hypothyroidism and hyperthyroidism with an increased risk of Parkinson disease. However, most of evidences in above literature regarding the causal relationship of TDs patients with MDs are from observational studies, which illustrate several shortcomings for causal inference, and it is the imminent challenge to understand whether MDs have effects of TDs by utilizing a technical tool without confounders. Hence, it is important to explore causal relationships between MDs and TDs to avoid shortcomings of previous studies.

Mendelian randomization (MRs) is an approach that establish causal relationships between exposure dataset and outcome dataset by utilizing single-nucleotide polymorphisms (SNPs), a kind of genetic variations, as instrumental variables (IVs).^[18]^ In the current study, we performed a two-sample MR study to explore causal relationships of TDs and MDs by utilizing IVs. Our study aims to find novel insights and empirical evidence of the field on causal relationships of TDs and MDs. Meanwhile, our MR analysis findings can provide fresh perspectives of the underlying pathogenesis and treatment of MDs and TDs.

## 2. Materials and methods

### 2.1. Data sources

In this paper, we selected 5 MDs, including Alzheimer disease, bipolar disorder, MDD, Parkinson disease, schizophrenia, as exposure dataset and 2 TDs (hyperthyroidism/thyrotoxicosis, hypothyroidism) as outcome dataset from genome-wide association studies (GWAS) summary data (portal: https://gwas.mrcieu.ac.uk/). The consortium of GWAS are Alzheimer Disease Genetics Consortium (ADGC), European Alzheimer’s Disease Initiative (EADI), Cohorts for Heart and Aging Research in Genomic Epidemiology Consortium (CHARGE), Genetic and Environmental Risk in AD/Defining Genetic, Polygenic and Environmental Risk for Alzheimer’s Disease Consortium (GERAD/PERADES), PGC Bipolar Disorder Working Group of the Psychiatric Genomics Consortium, Psychiatric Genomics Consortium (PGC), International Parkinson’s Disease Genomics Consortium (IPDGC) and Medical Research Council Integrative Epidemiology Unit (MRC-IEU). Our MR study leveraged publicly available GWAS data. Hence, we do not need to find patient consent and ethics committee approval. The detailed information of GWAS dataset is presented in Table 1.

**Table 1:** The detailed GWAS data information of MR study on causal relationships of MDs and TDs.

Type	Consortium	Population	Sample size	N_case	N_control	ID in GWAS
*Exposure*						
Alzheimer disease	ADGC et al	European	63,926	21,982	41,944	ieu-b-2
Bipolar disorder	PGC	European	413,466	41,917	371,549	ieu-b-5110
MDD	PGC	European	480,359	135,458	344,901	ieu-a-1187
Parkinson disease	IPDGC	European	482,730	33,674	449,056	ieu-b-7
Schizophrenia	PGC	Mixed	82,315	35,476	46,839	ieu-a-22
*Outcome*						
Hyperthyroidism/thyrotoxicosis	MRC-IEU	European	462,933	3545	459,388	ukb-b-20289
Hypothyroidism	MRC-IEU	European	463,010	9674	453,336	ukb-b-4226

GWAS = Genome-wide association studies, MD = mental disease, MDD = major depressive disorder, MR = Mendelian randomization, TD = thyroid disease.

### 2.2. Genetic instruments selection

In the current study, MDs served as exposure dataset, whereas TDs served as outcome dataset. In order to make sure the effectiveness of our MR analysis conclusions, the *P* value of SNPs was set as less than $5\times 10^{-8}$, and the clumping process with $r^{2}<0.001$ and *kb* = 10,000 were utilized to assess linkage disequilibrium of SNPs relationships.^[19]^ Meanwhile, there was no linkage disequilibrium among each SNP when screening IVs.^[20]^ *F*-statistic^[21]^ was computed as followed:

$$\begin{array}{l} F=\frac{N-K-1}{K}\times \frac{R^{2}}{1-R^{2}} \end{array}$$ (1)

where $R^{2}$ represents the degree of exposure based on IVs, N represents size of samples, K represents included SNPs numbers, for each SNPs, the value of K is 1.^[22]^ Then the calculation approach of w is discussed as followed. If the value of minor allele frequency (MAF) is existing, $R^{2}=2\times MAF\times \left(1-MAF\right)\times \beta^{2}$, where β represents the effect of SNP on exposure. If the value of MAF is not existing but effect allele frequency (EAF) can be obtained, $R^{2}=2\times EAF\times \left(1-EAF\right)\times \beta^{2}$. In addition, if EAF>0.5, then MAF=1−EAF. If EAF≤0.5, then MAF=EAF. So MAF×(1−MAF) and EAF×(1−EAF) are symmetric. If both values of MAF and EAF are not existing, $R^{2}=\frac{2\times EAF\times \left(1-EAF\right)\times \beta^{2}}{2\times EAF\times \left(1-EAF\right)\times \beta^{2}+2\times SE^{2}\times N\times EAF\times \left(1-EAF\right)}$, where SE is the standard error of β. It is obvious that EAF×(1−EAF) did not participate in the calculation of $R^{2}$. For F<10, SNPs were considered as weak instruments and were supposed to be excluded.^[23]^

### 2.3. Two-sample MR statistical analysis

In the present study, the following 3 core assumptions were satisfied for the two-sample MR method.^[24,25]^ Initially, IVs screened from GWAS were associated with MDs. Subsequently, the IVs screened did not have causal relationships with any unknown confounders of MDs. Finally, IVs did not have causal relationships with TDs through MDs, but not in other ways. For regression evaluation methods, the inverse variance weighted (IVW)^[26]^ was considered as the primary MR analysis method.^[27]^ Meanwhile, IVW (fixed effects),^[26]^ maximum likelihood,^[26]^ MR Egger,^[28]^ weighted median, penalized weighted median,^[29]^ simple mode and weighted mode^[30]^ were also utilized for features. MR-Egger and IVW test regression were employed to examine horizontal pleiotripy and heterogeneity, respectively. MR-PRESSO global test was performed to identify and remove outliers to detect horizontal pleiotropy. The significant threshold parameter of MR-PRESSO was set as 0.05 and seed number was set as 2000. All of MR statistical analyzes were based on R v.4.3.1, TwoSampleMR v.0.5.7^[31]^ and MR-PRESSO package v.1.0.^[32]^

## 3. Results

### 3.1. Information of instrumental variables

According to set parameters ($p<5\times 10^{-8}$, $r^{2}=0.001$, kb = 10,000, F >10), 21 SNPs in Alzheimer diseases, 52 SNPs in bipolar disorder, 36 SNPs in MDD, 23 SNPs in Parkinson diseases and 83 SNPs in schizophrenia are obtained for further analysis. We harmonized MDs and TDs with excluding SNPs for being palindromic with intermediate allele frequencies. For hyperthyroidism/thyrotoxicosis, 1 SNPs in Alzheimer disease (rs11257242), 7 SNPs in bipolar disorder (rs10455979, rs10791849, rs11062170, rs13417268, rs1998820, rs2011302, rs62489493), 2 SNPs in MDD (rs34215985, rs62099069), 1 SNPs in Parkinson disease (rs10451230) and 9 SNPs in schizophrenia (rs11139497, rs11191419, rs11740474, rs12325245, rs215411, rs2332700, rs281768, rs2851447, rs9607782) are removed. For hypothyroidism, 1 SNPs in Alzheimer disease (rs11257242), 7 SNPs in bipolar disorder (rs10455979, rs10791849, rs11062170, rs13417268, rs1998820, rs2011302, rs62489493), 2 SNPs in MDD (rs34215985, rs62099069), 1 SNPs in Parkinson disease (rs10451230) and 9 SNPs in schizophrenia (rs11139497, rs11191419, rs11740474, rs12325245, rs215411, rs2332700, rs281768, rs2851447, rs9607782) are removed.

### 3.2. Two-sample MR analysis between mental diseases and thyroid diseases

#### 3.2.1. Causal relationships of Alzheimer disease and thyroid diseases

**Figure 1.**
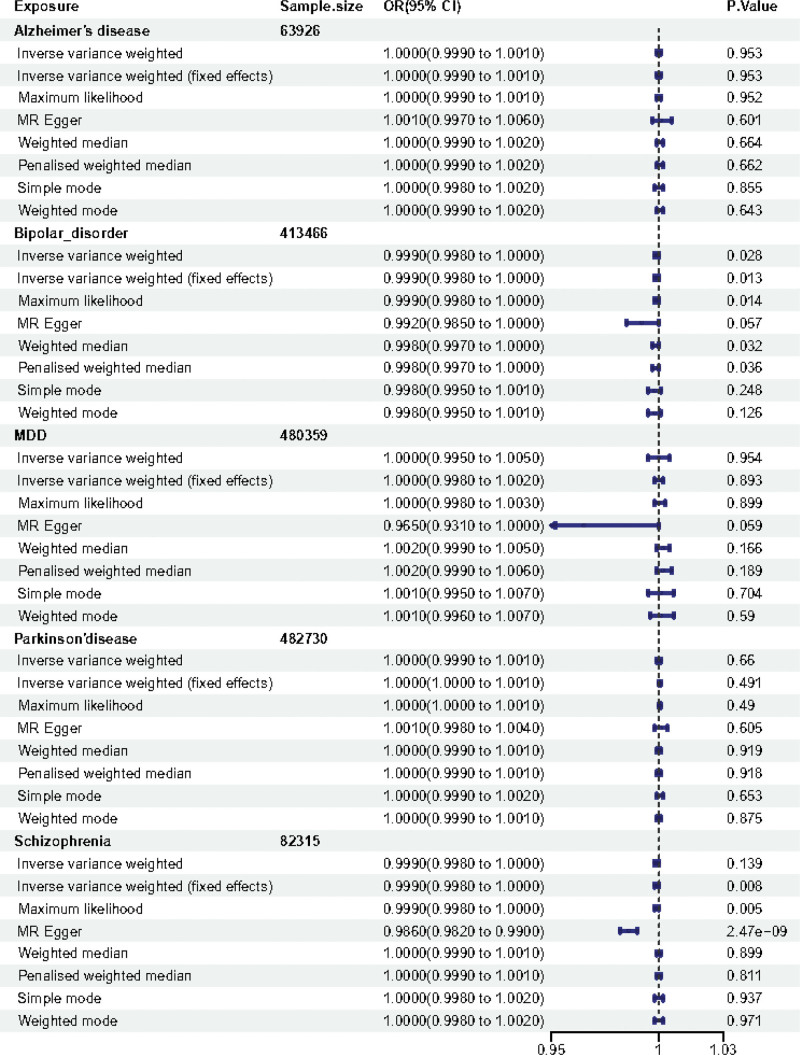
Associations of genetically predicted MDs of hyperthyroidism/thyrotoxicosis. The forest plot of causal relationship between MDs and hyperthyroidism/thyrotoxicosis based on 8 MR methods. CI = confidence interval; MD = mental disease; MR = Mendelian randomization; OR = odds ratio.

**Figure 2.**
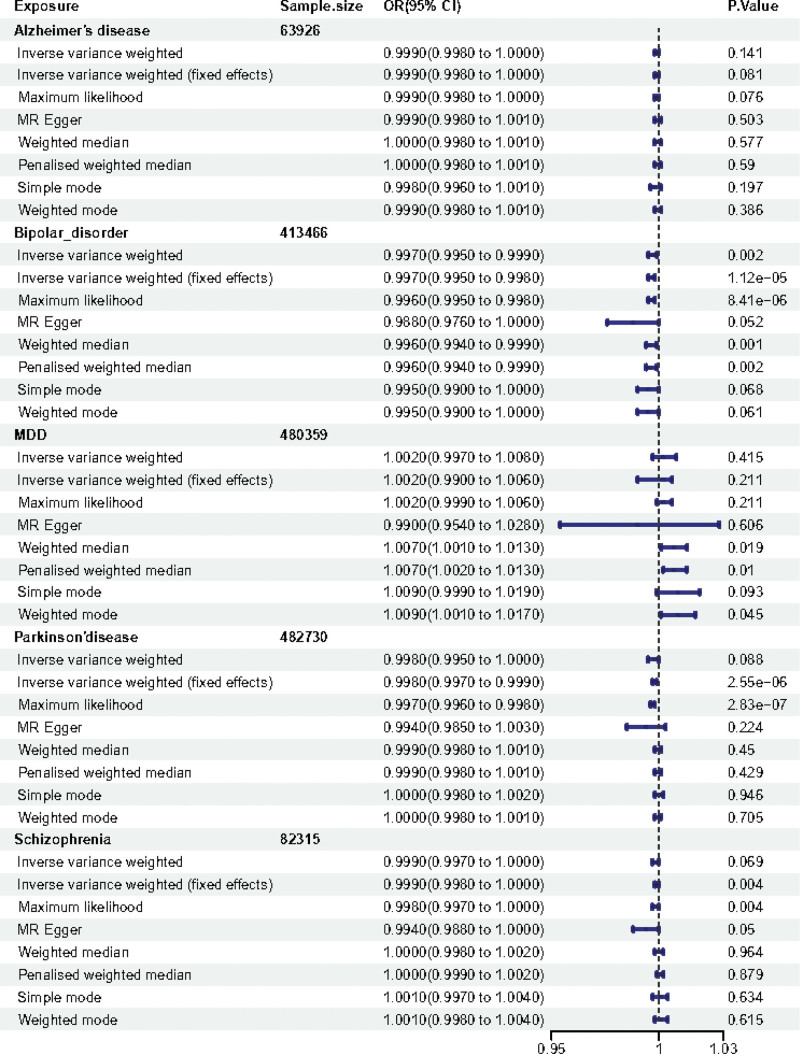
Associations of genetically predicted MDs of hypothyroidism. The forest plot of causal relationship between MDs and hypothyroidism based on 8 MR methods. CI = confidence interval; MD = mental disease; MR = Mendelian randomization; OR = odds ratio.

For the hyperthyroidism/thyrotoxicosis, Alzheimer disease showed no MR association [IVW: odds ratio [OR] (95% confidence interval [CI]) 1.000 (0.999–1.001), *P* = .953; IVW (fixed effects): OR (95% CI) 1.000 (0.999–1.001), *P* = .953; maximum likelihood [ML]: OR (95% CI) 1.000 (0.999–1.001), *P* = .952; MR Egger: OR (95% CI) 1.001 (0.997–1.006), *P* = .601; weighted median: OR (95% CI) 1.000 (0.999–1.002), *P* =.664; penalised weighted median: OR (95% CI) 1.000 (0.999–1.002), *P* = .662; simple mode: OR (95% CI) 1.000 (0.998–1.002), *P* = .855; weighted mode: OR (95% CI) 1.000 (0.999–1.002), *P* = .643], as shown in Figure 1. For the hypothyroidism, Alzheimer disease showed no MR association [IVW: OR (95% CI) 0.999 (0.998–1.000), *P* = .141; IVW (fixed effects): OR (95% CI) 0.999 (0.998–1.000), *P* = .081; ML: OR (95% CI) 0.999 (0.998–1.000), *P* = .076; MR Egger: OR (95% CI) 0.999 (0.998–1.001), *P* = .503; weighted median: OR (95% CI) 1.000 (0.998–1.001), *P* = .577; penalised weighted median: OR (95% CI) 1.000 (0.998–1.001), *P* = .59; simple mode: OR (95% CI) 0.998 (0.996–1.001), *P* = .197; weighted mode: OR (95% CI) 0.999 (0.998–1.001), *P* = .386], as shown in Figure 2.

#### 3.2.2. Causal relationships of bipolar disorder and thyroid diseases

For the hyperthyroidism/thyrotoxicosis, interestingly, the results of IVW analysis method displayed that causal relationships of bipolar disorder and the risk of hyperthyroidism/thyrotoxicosis is statistically significant in IVW method with *P* < .05 [IVW: OR (95% CI) 0.999 (0.998–1.000), *P* = .028; IVW(fixed effects): OR (95% CI) 0.999 (0.998–1.000), *P* = .013; ML: OR (95% CI) 0.999 (0.998–1.000), *P* = .014; MR Egger: OR (95% CI) 0.992 (0.985–1.000), *P* = .057; weighted median: OR (95% CI) 0.998 (0.997–1.000), *P* = .032; penalised weighted median: OR (95% CI) 0.998 (0.997–1.000), *P* = .036; simple mode: OR (95% CI) 0.998 (0.995–1.001), *P* = .248; weighted mode: OR (95% CI) 0.998 (0.995–1.001), *P* = .126], as shown in Figure 1. For the hypothyroidism, the results of IVW analysis method displayed that causal relationships of bipolar disorder and the risk of hypothyroidism is also statistically significant in IVW method with *P* < 0.05 [IVW: OR (95% CI) 0.997 (0.995–0.999), *P* = .002; IVW (fixed effects): OR (95% CI) 0.997 (0.995–0.998), *p* = 1.12E‐05; ML: OR (95% CI) 0.996 (0.995–0.998), *P* = 8.41E‐06; MR Egger: OR (95% CI) 0.988 (0.976–1.000), *P* = .052; weighted median: OR (95% CI) 0.996 (0.994–0.999), *P* = .001; penalised weighted median: OR (95% CI) 0.996 (0.994–0.999), *P* = .002; simple mode: OR (95% CI) 0.995 (0.990–1.000), *P* = .068; weighted mode: OR (95% CI) 0.995 (0.990–1.000), *P* = .061], as shown in Figure 2.

#### 3.2.3. Causal relationships of MDD and thyroid diseases

For the hyperthyroidism/thyrotoxicosis, MDD showed no MR association [IVW: OR (95% CI) 1.000 (0.995–1.005), *P* = .954; IVW (fixed effects): OR (95% CI) 1.000 (0.998–1.002), *P* = .893; ML: OR (95% CI) 1.000 (0.998–1.003), *P* = .899; MR Egger: OR (95% CI) 0.965 (0.931–1.000), *P* = .059; weighted median: OR (95% CI) 1.002 (0.999–1.005), *P* = .166; penalised weighted median: OR (95% CI) 1.002 (0.999–1.006), *P* = .189; simple mode: OR (95% CI) 1.001 (0.995–1.007), *P* = .704; weighted mode: OR (95% CI) 1.001 (0.996–1.007), *P* = .59], as shown in Figure 1. For the hypothyroidism, MDD showed no MR association [IVW: OR (95% CI) 1.002 (0.997–1.008), *P* = .415; IVW (fixed effects): OR (95% CI) 1.002 (0.990–1.006), *P* = .211; ML: OR (95% CI) 1.002 (0.999–1.006), *P* = .211; MR Egger: OR (95% CI) 0.99 (0.954–1.028), *P* = .606; weighted median: OR (95% CI) 1.007 (1.001–1.013), *P* = .019; penalised weighted median: OR (95% CI) 1.007 (1.002–1.013), *P* = .01; simple mode: OR (95% CI) 1.009 (0.999–1.019), *P* = .093; weighted mode: OR (95% CI) 1.009 (1.001–1.017), *P* = .045], as shown in Figure 2.

#### 3.2.4. Causal relationships of Parkinson disease and thyroid diseases

For the hyperthyroidism/thyrotoxicosis, Parkinson disease showed no MR association [IVW: OR (95% CI) 1.000 (0.999–1.001), *P* = .66; IVW (fixed effects): OR (95% CI) 1.000 (1.000–1.001), *P* = .491; ML: OR (95% CI) 1.000 (1.000–1.001), *P* = .49; MR Egger: OR (95% CI) 1.001 (0.998–1.004), *P* = .605; weighted median: OR (95% CI) 1.000 (0.999–1.001), *P* = .919; penalised weighted median: OR (95% CI) 1.000 (0.999–1.001), *P* = .918; simple mode: OR (95% CI) 1.000 (0.999–1.002), *P* = .653; weighted mode: OR (95% CI) 1.000 (0.999–1.001), *P* = .875], as shown in Figure 1. For the hypothyroidism, Parkinson disease showed no MR association [IVW: OR (95% CI) 0.998 (0.995–1.000), *P* = .088; IVW (fixed effects): OR (95% CI) 0.998 (0.997–0.999), *p* = 2.55E‐06; ML: OR (95% CI) 0.997 (0.996–0.998), *p* = 2.83E‐07; MR Egger: OR (95% CI) 0.994 (0.985–1.003), *P* = .224; weighted median: OR (95% CI) 0.999 (0.998–1.001), *P* = .45; penalised weighted median: OR (95% CI) 0.999 (0.998–1.001), *P* = .429; simple mode: OR (95% CI) 1.000 (0.998–1.002), *P* = .946; weighted mode: OR (95% CI) 1.000 (0.998–1.001), *P* = .705], as shown in Figure 2.

#### 3.2.5. Causal relationships of schizophrenia and thyroid diseases

For the hyperthyroidism/thyrotoxicosis, schizophrenia showed no MR association [IVW: OR (95% CI) 0.999 (0.998–1.000), *P* = .139; IVW (fixed effects): OR (95% CI) 0.999 (0.998–1.000), *P* = .008; ML: OR (95% CI) 0.999 (0.998–1.000), *P* = .005; MR Egger: OR (95% CI) 0.986 (0.982–0.990), *p* = 2.47E‐09; weighted median: OR (95% CI) 1.000 (0.999–1.001), *P* = .899; penalised weighted median: OR (95% CI) 1.000 (0.999–1.001), *P* = .811; simple mode: OR (95 % CI) 1.000 (0.998–1.002), *P* = .937; weighted mode: OR (95% CI) 1.000 (0.998–1.002), *P* = .971], as shown in Figure 1. For the hypothyroidism, schizophrenia showed no MR association [IVW: OR (95% CI) 0.999 (0.997–1.000), *P* = .069; IVW (fixed effects): OR (95% CI) 0.999 (0.998–1.000), *P* = .004; ML: OR (95% CI) 0.998 (0.997–1.000), *P* = .004; MR Egger: OR (95% CI) 0.994 (0.988–1.000), *P* = .05; weighted median: OR (95% CI) 1.000 (0.998–1.002), *P* = .964; penalised weighted median: OR (95% CI) 1.000 (0.999–1.002), *P* = .879; simple mode: OR (95% CI) 1.001 (0.997–1.004), *P* = .634; weighted mode: OR (95% CI) 1.001 (0.998–1.004), *P* = .615], as shown in Figure 2.

### 3.3. Robustness analysis

In the current study, MR-PRESSO method was used to remove outliers for further robustness analysis.^[33]^ For robustness analysis, Cochran Q test^[34]^ was used to consider the effectiveness of heterogeneity, which can be calculated as:

$$\begin{array}{l} Q=\underset{i}{\sum }\;w_{i}{\left({\hat{\beta }}_{i}^{IV}-\beta_{i}^{IV}\right)}^{2} \end{array}$$ (2)

where $w_{i}$ is the weight to estimate ${\hat{\beta }}_{i}^{IV}$, and $\beta_{i}^{IV}$ is a weighted mean estimate, which can be computed as $\beta_{i}^{IV}=\sum w_{i}{\hat{\beta }}_{i}^{IV}/\sum w_{i}$.^[35]^ The detailed information of heterogeneity and pleiotropy between MDs and TDs were shown in Table 2. In this table, MR analysis of MDD and the risk of hyperthyroidism/thyrotoxicosis had significant heterogeneity (*P* < .05) with p=4.43E−18, MDD and the risk of hypothyroidism had significant heterogeneity (*P* < .05) with *P* = .022 and schizophrenia and the risk of hypothyroidism also had significant heterogeneity (*P* < .05) with *P* = .0009. For horizontal pleiotropy, there were no evidences that causal relationships of MDs and TDs had horizontal pleiotropy (*P* < .05) based on MR-Egger method.^[36]^ More details about robustness analysis, such as leave-one-out analysis, funnel plot and scatter plot between MDs and TDs, were shown in Figure S1, Supplemental Digital Content, http://links.lww.com/MD/M745 and Figure S2, Supplemental Digital Content, http://links.lww.com/MD/M746.

**Table 2:** IVW heterogeneity test and MR-Egger pleiotripy test between mental diseases and thyroid diseases.

Exposure	Outcome	IVW heterogeneity test		MR-Egger pleiotropy test	
		Q	*P* value	Intercept	*P* value
Alzheimer disease	Hyperthyroidism/thyrotoxicosis	9.954	.444	‐0.0001	.601
	Hypothyroidism	16.925	.152	‐6.70E‐05	.695
Bipolar disorder	Hyperthyroidism/thyrotoxicosis	51.554	.104	.0004	.102
	hypothyroidism	53.131	.097	.0005	.166
MDD	Hyperthyroidism/thyrotoxicosis	144.830	4.43E-18	0.001	.055
	Hypothyroidism	43.659	.022	‐0.0003	.534
Parkinson disease	Hyperthyroidism/thyrotoxicosis	12.406	.648	0.0001	.454
	Hypothyroidism	12.878	.682	7.75E‐05	.773
Schizophrenia	Hyperthyroidism/thyrotoxicosis	57.573	.601	0.0001	.522
	Hypothyroidism	105.064	.0009	‐0.0002	.533

IVW = inverse variance weighted, MDD = major depressive disorder, MR = Mendelian randomization.

## 4. Discussion

The current MR statistical analysis was performed to investigate causal relationships between MDs (Alzheimer disease, bipolar disorder, MDD, Parkinson disease, schizophrenia) and the risk of TDs (hyperthyroidism/thyrotoxicosis, hypothyroidism). To the best of our knowledge, it is the first MR analysis to explore whether MDs are causally related to TDs. The IVW regression method confirmed that there is a causal relationship between bipolar disorder and the risk of TDs. For other MDs, no causal evidences were found with residual MDs (Alzheimer disease, MDD, Parkinson disease, schizophrenia).

Some studies have shown causal associations between TDs and MDs. In,^[37]^ this study has suggested that hyperthyroidism has possibly clinical factors to increase the risk of Alzheimer disease in elderly persons. Our study found that hyperthyroidism was not related to Alzheimer disease (IVW: OR [95% CI] 0.999 [0.998–1.000], *P* = .141). However, a recent study showcases that hyperthyroidism can affect dementia of the Alzheimer disease risk in elderly persons, thus increased levels of T4 and free T4 elevate the risk of developing Alzheimer disease in patients.^[38]^ A previous case–control study has reported that patients diagnosed with both bipolar affective disorder and hyperthyroidism have symptoms of mania. Meanwhile, levels of triiodothyronine hormone in serum (thyroid dysfunction) and bipolar affective disorder have significant associations based on the statistical analysis.^[39]^ This clinical result is consistent with the conclusions of our study for our statistical analysis between bipolar disorder and TDs. For MDD, a recent study found out that hypothyroidism or subclinical changes were statistically correlated with MDD patients and the severity of depression.^[40]^ In,^[41]^ authors leveraged statistical methods to validate differences in the prevalence of hypothyroidism and hyperthyroidism between patients with MDD and the general population based on the National Health Research Institute database, but our study demonstrated that there were no associations between MDD and TDs [hyperthyroidism/thyrotoxicosis: IVW: OR (95% CI) 1.000 (0.995–1.005), *P* = .954. Hypothyroidism: IVW: OR (95% CI) 1.002 (0.997–1.008), *P* = .415]. The reason may be the difference of population samples. Furthermore, a previous population-based research found out that patients with schizophrenia is significantly more predominant in patients diagnosed with hypothyroid compared to controls.^[42]^ It has been reported that the levels of TSH might increase the risk of schizophrenia.^[43]^

Previous researches revealed the causal link between thyroid function with normal-range thyrotropin (TSH) and free thyroxine levels and schizophrenia.^[44]^ In,^[45]^ a MR study revealed that causal relationships of increased levels of genetically predicted TSH within the normal range and in younger individuals, full range TSH and free thyroxine or hypothyroidism and Alzheimer disease. The research in^[46]^ demonstrated that higher free thyroxine level showed a protective role on bipolar disorder risk based on a two-sample MR analysis. For MDs,^[47]^ changes in reference-range TSH and free thyroxine levels have no influence on the risk of MDD, and neither on minor depressive symptoms.

Moreover, it needs to be emphasized that our MR study still has some constraints. Firstly, our understanding of TDs (e.g., hypothyroidism, hyperthyroidism/thyrotoxicosis) and MDs (e.g., Alzheimer disease, bipolar disorder, MDD, Parkinson disease, schizophrenia) is limited. Hence, two-sample MR analysis may generate biased results. Meanwhile, we failed to utilize a bidirection MR analysis to explore a reverse causality association between MDs and TDs, and it needs to be leveraged for further analysis. Secondly, the threshold of our IVs (*P* value, F statistic) is effect but we did not utilize Bonferroni-corrected method and remove false negative errors. Future investigations are supposed to employ more non-European populations as the source of GWAS dataset. Finally, the absence of publicly GWAS dataset eliminates our study from determining the underlying sample overlap bias. For future studies, subsequently clinical trials will be necessary to validate the potential pathogenesis, development and treatment of MDs and TDs.

## 5. Conclusions

To sum up, our two-sample MR study confirmed that bipolar disorder had direct causal relationships with TDs. For other MDs, our study did not provide enough evidences to support that MDs had causal links with TDs. Possible reasons may be observational studies were confoundered by other unknown elements. It is significant that subsequent researches will need to explore causal relationships between MDs and TDs, and fully understand the causal link between MDs and the risk of TDs for the subsequent treatment.

## Acknowledgments

We would like to thank supports from the First Affiliated Hospital of China University of Science and Technology (Anhui Provincial Hospital), Hefei Second People’s Hospital and Anhui University of Chinese Medicine and the contributions from the participants in our study. For the data used in these analyses, we gratefully acknowledge the IEU open GWAS project (https://gwas.mrcieu.ac.uk).

## Author contributions

**Conceptualization:** Xiang Fang, Cuiping Wu, Wenjing Ding, Zhangxia Shi.

**Investigation:** Xiang Fang, Dandan Xu.

**Methodology:** Xiang Fang, Cuiping Wu, Wenjing Ding, Zhangxia Shi.

**Writing – original draft:** Xiang Fang, Cuiping Wu.

**Writing – review & editing:** Xiang Fang, Wenjing Ding, Dandan Xu, Zhangxia Shi.

## Supplementary Material




