# Supplementary material for: Causal relationships of mental diseases and thyroid diseases based on a Mendelian randomization study
Source: Medicine (Baltimore). 2024 May 31;103(22):e38223. doi: 10.1097/MD.0000000000038223 (PMC11142779; doi:10.1097/MD.0000000000038223)

Figure S2. Leave-one-out analysis, scatter plot, forest plot, and funnel plot between mental diseases (Alzheimer's disease, bipolar disorder, major depressive disorder, Parkinson's disease, schizophrenia) and hypothyroidism.

1. Leave-one-out analysis, scatter plot, forest plot, and funnel plot between Alzheimer's disease and hypothyroidism


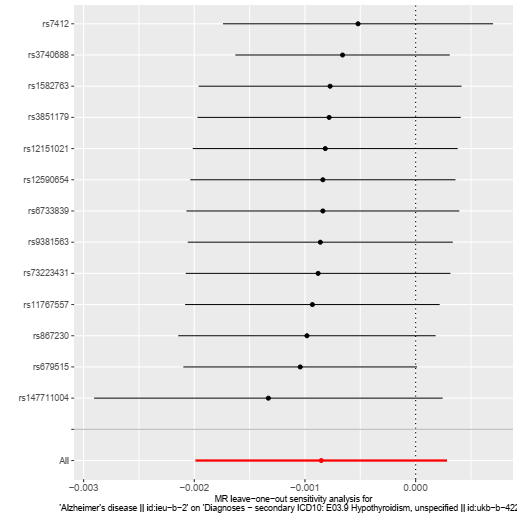


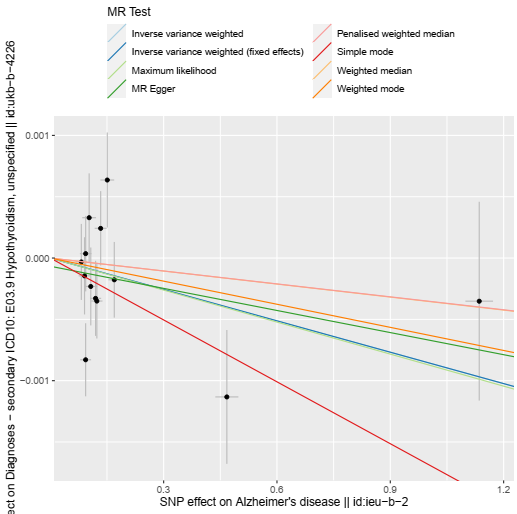


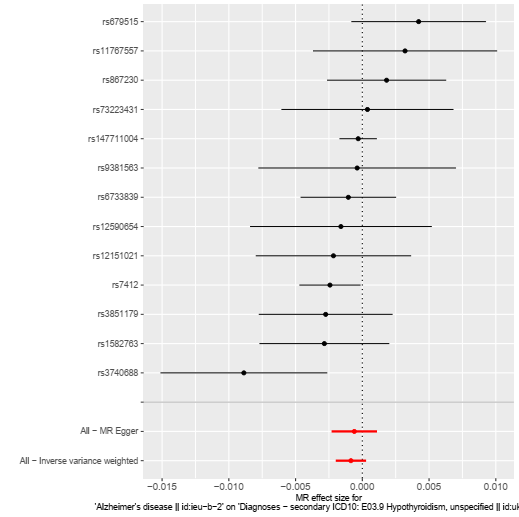


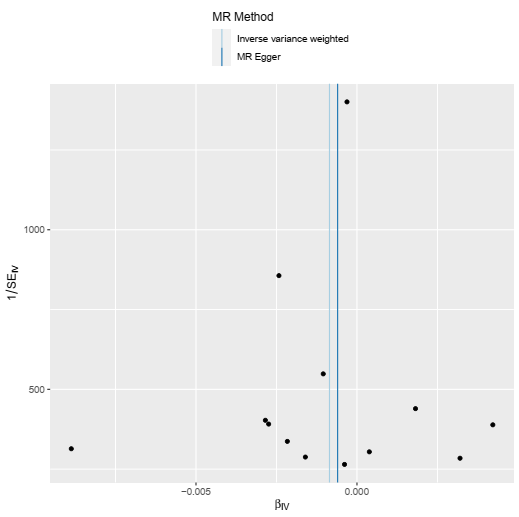


1. Leave-one-out analysis, scatter plot, forest plot, and funnel plot between bipolar disorder and hypothyroidism


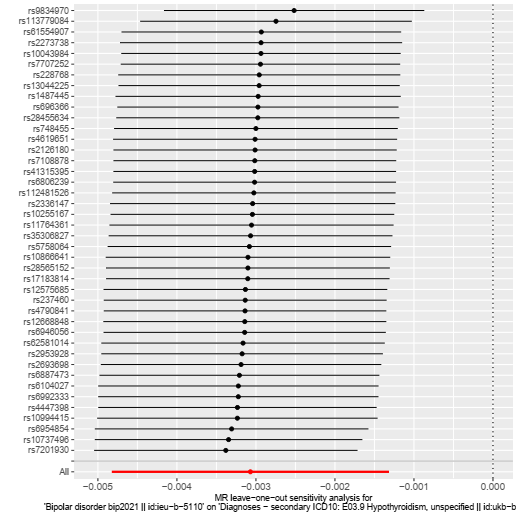


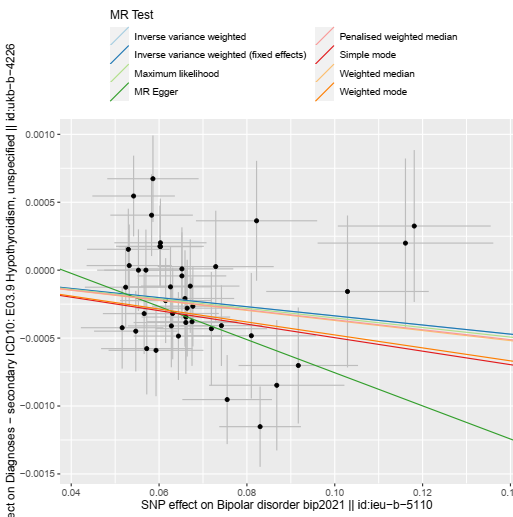


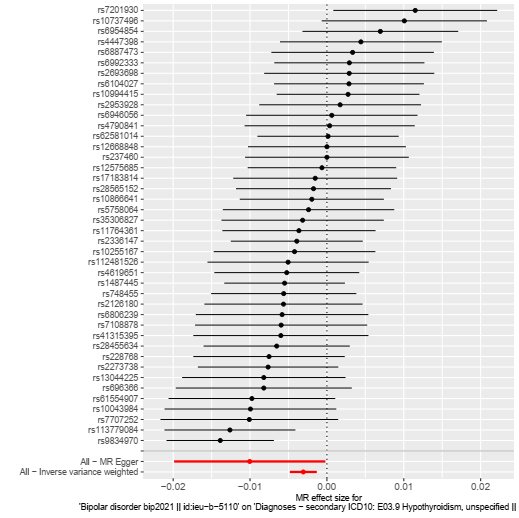


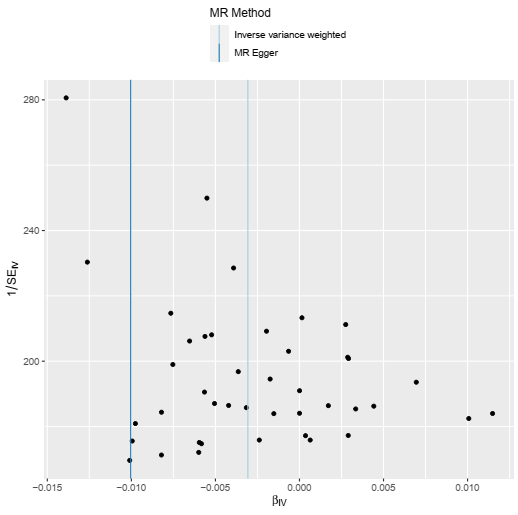


1. Leave-one-out analysis, scatter plot, forest plot, and funnel plot between major depressive disorder and hypothyroidism


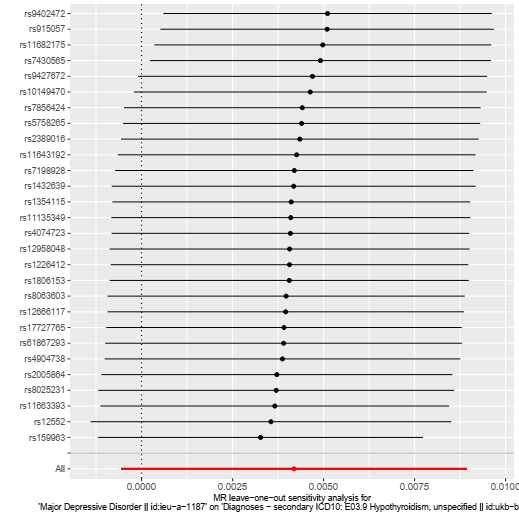


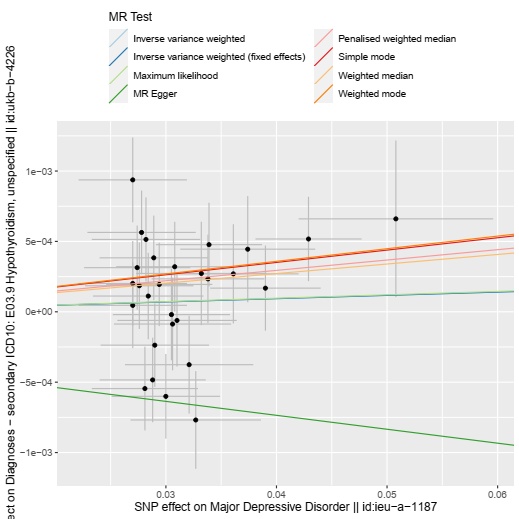


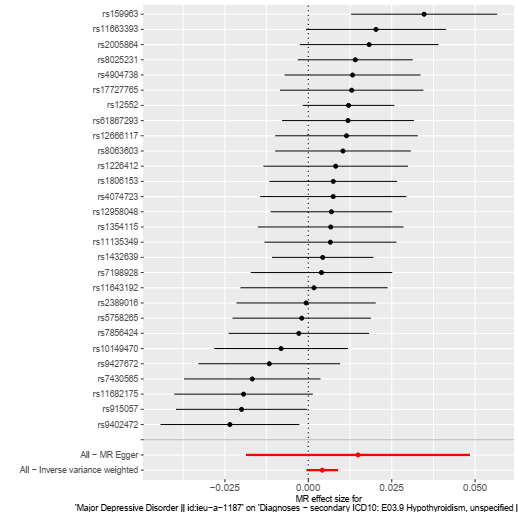


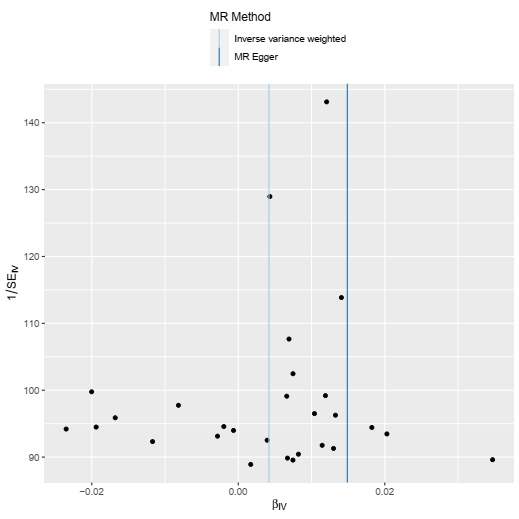


1. Leave-one-out analysis, scatter plot, forest plot, and funnel plot between Parkinson's disease and hypothyroidism


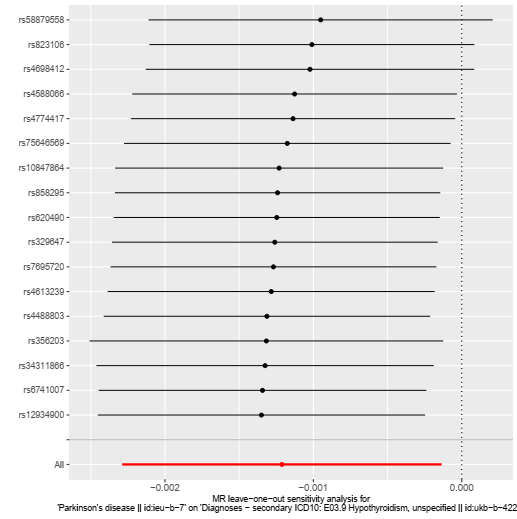


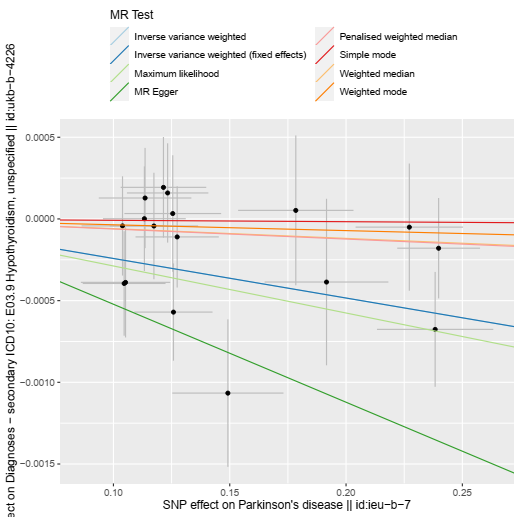


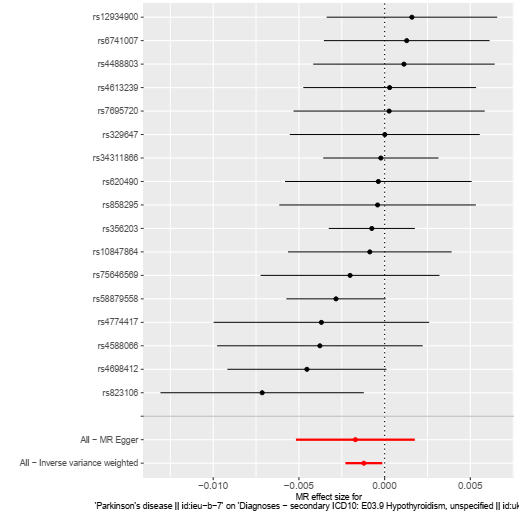


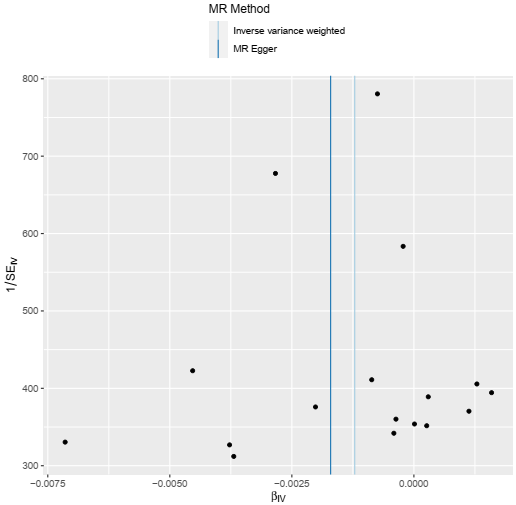


1. Leave-one-out analysis, scatter plot, forest plot, and funnel plot between schizophrenia and hypothyroidism


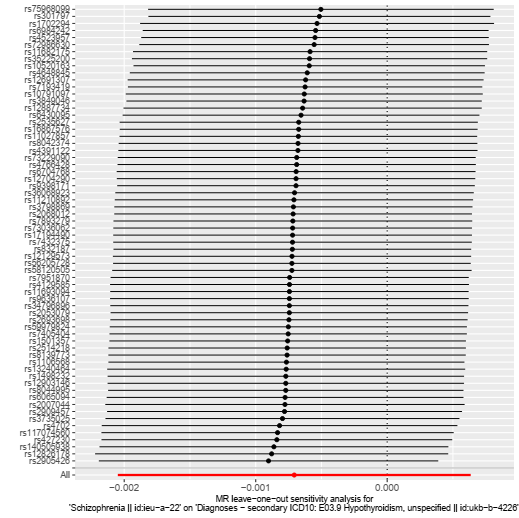


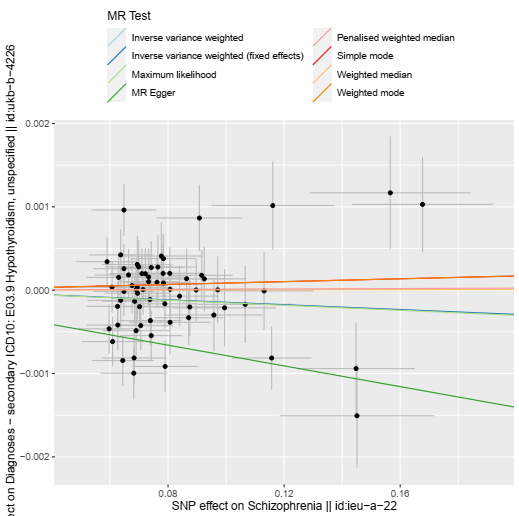


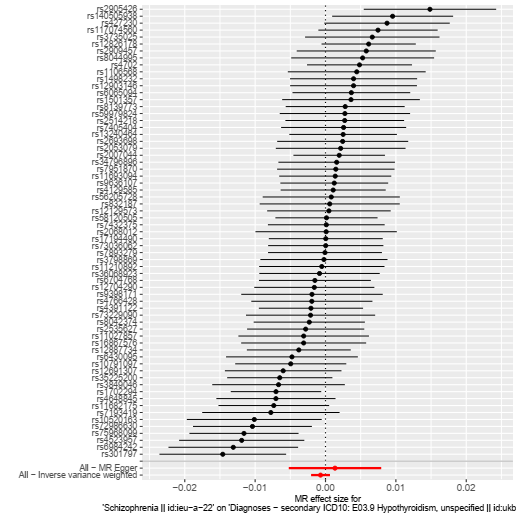


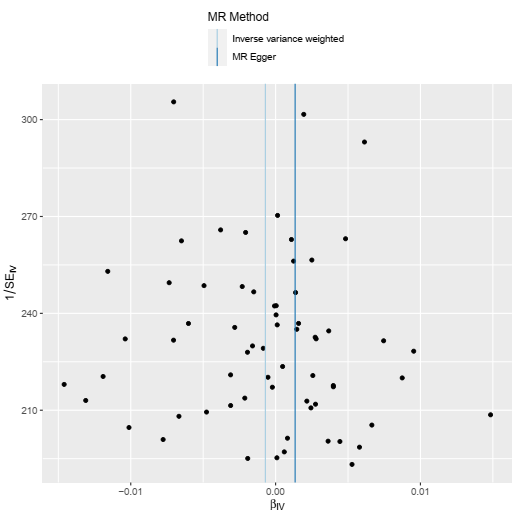

Supplement: Supplementary file 2 [file medi-103-e38223-s002.docx]
